# Post-migration HIV acquisition: A systematic review and meta-analysis

**DOI:** 10.1017/S0950268824000372

**Published:** 2024-03-01

**Authors:** Simran Mann, Zeenathnisa Mougammadou, Jan Wohlfahrt, Rahma Elmahdi

**Affiliations:** 1 School of Public Health, Imperial College London, London, UK; 2 Preventive Medicine, National University Hospital, Singapore; 3 The Danish Cancer Society, Copenhagen, Denmark; 4 Department of Clinical Medicine, Aalborg University, Copenhagen, Denmark; 5 Department of Gastroenterology and Hepatology, Aalborg University Hospital, Aalborg, Denmark

**Keywords:** epidemiology, HIV, migrant, public health, migration

## Abstract

Migrants in Europe face a disproportionate burden of HIV infection; however, it remains unclear if this can be prevented through public health interventions in host countries. We undertake a systematic review and meta-analysis to estimate post-migration HIV acquisition (PMHA) as a proportion of all HIV cases in European migrants. MEDLINE, EMBASE, Global Health, HMIC, and Cochrane Library were searched with terms capturing ‘HIV’, ‘migration’, and ‘Europe’. Data relating to the proportion of HIV acquired following migration were extracted and random-effects model (REM) meta-analysis was undertaken to calculate a pooled estimate for the proportion of PMHA in European countries. Subgroup meta-analysis was undertaken for PMHA by migrant demographic characteristics and host country. Fifteen articles were included for systematic review following retrieval and screening of 2,320 articles. A total of 47,182 migrants in 11 European countries were included in REM meta-analysis, showing an overall PMHA proportion of 0.30 (95% CI: 0.23–0.38). Subgroup analysis showed no significant difference in PMHA between host country and migrant demographic characteristics. This work illustrates that migrants continue to be at high risk of HIV acquisition in Europe. This indicates the need for targeted screening and HIV prevention interventions, ensuring resources are appropriately directed to combat the spread of HIV.

## Background

Migrant populations in Europe bear a disproportionate burden of HIV, accounting for more than one-third of all newly diagnosed HIV cases in the EU/EEA [1]. Despite being a significant risk group for HIV, migrants are often diagnosed at a later stage than non-migrants and have lower uptake of prevention or treatment interventions [2,3]. Furthermore, migrants have been shown to have poorer outcomes at each stage of the HIV continuum of care, including later diagnoses, lower rates of adherence to treatment, and lower rates of viral suppression when compared with non-migrant populations [4,5].

When combating the burden of HIV in migrant populations, the focus is placed on screening migrants on entry to ensure the linkage of migrants living with HIV into care in their host country [1–3]. The focus on screening as prevention stems from the assumption that HIV is most often acquired in the country of origin, before or during migration. However, current literature suggests that a large proportion of HIV cases in migrant populations are acquired post-migration (i.e., in the host country) [4–6]. Therefore, efforts to identify HIV only in recent migrants could be rendered insufficient to prevent the spread of HIV as they may not protect vulnerable patients within migrant groups from HIV acquisition post-migration.

An accurate estimate of PMHA is key to addressing disparities in migrant access to HIV prevention and treatment. Such estimates play a vital role in identifying opportunities to effectively eliminate these gaps by identifying new clusters of HIV infections for targeted screening and treatment programmes or it could identify subgroups with very high HIV acquisition risks. This could inform national and local prevention measures, including education of health risks and provision of pre-exposure prophylaxis to high-risk groups within migrant populations. Such an insight will also guide clinicians’ views and reframe the approach to preventative measures for high-risk groups within migrant populations.

Of note, the aMASE study and the PARCOURS study attempted to quantify post-migration HIV acquisition (PMHA) in migrant populations [5, 6]. However, PARCOURS, which classified 35% of HIV cases as PMHA, was based solely in France. aMASE estimated PMHA to be 63%, but used a ‘convenience sample’ across Europe, limiting the study’s external validity. Although these studies provide good insight into PMHA, they do not provide sufficient evidence from different migrant groups in different countries to estimate the average proportion of PMHA across Europe.

The aim of this study therefore was to quantify PMHA, as a proportion of all HIV cases in migrants in Europe through systematic review and meta-analysis of the published data. The primary outcome of this study was the proportion of PMHA in migrants in Europe; secondary outcomes were proportions of PMHA in migrant groups by reporting country, region of origin, gender, and sexuality. This study also intended to estimate PMHA by reporting country and by migrant demographic subgroup, including region of origin, gender, and sexuality.

## Methods

Migrants were classified in the inclusion process according to the UN definition: ‘a person who moves to a new country for a period of ≥1 year so the country of destination effectively becomes his/her new country of usual residence’. PMHA was defined as the acquisition of HIV infection and was determined from either i) ‘life events’ in the patient history of an HIV-positive migrant (i.e., a previous negative test taken post-migration indicates that the patient was HIV-negative on entry into the host country) or ii) matching patients’ CD4 count, or other reliable biomarker level, at diagnosis with a CD4 decline model in untreated HIV-infected patients. Matching with such a model allowed for the estimation of the infection date. If the date of infection occurred after migration, it was classified as PMHA.

### Search strategy

In August 2022, MEDLINE, EMBASE, Global Health, Health management information consortium (HMIC), and Cochrane Library were searched for studies fulfilling the inclusion criteria. Reference lists from included articles were subsequently screened for more articles for potential inclusion. Only published, peer-reviewed articles were included. Grey literature or conference abstracts were excluded to ensure the inclusion of only high-quality, peer-reviewed studies. Search terms and subject headings included migrant*, HIV, and Europ* (Supplementary materials Table 1: *Search Terms*).

All study types providing a quantitative estimate, including surveillance reports and mathematical modelling studies, were included. To account for the impact of antiretroviral therapy on HIV transmission pathways, the search was limited to articles published since 1996. Papers were excluded if they did not provide an estimate of average PMHA in first-generation migrant populations in a European country (Supplementary materials Table 2: *Screening Criteria*).

### Screening

**Figure 1.**
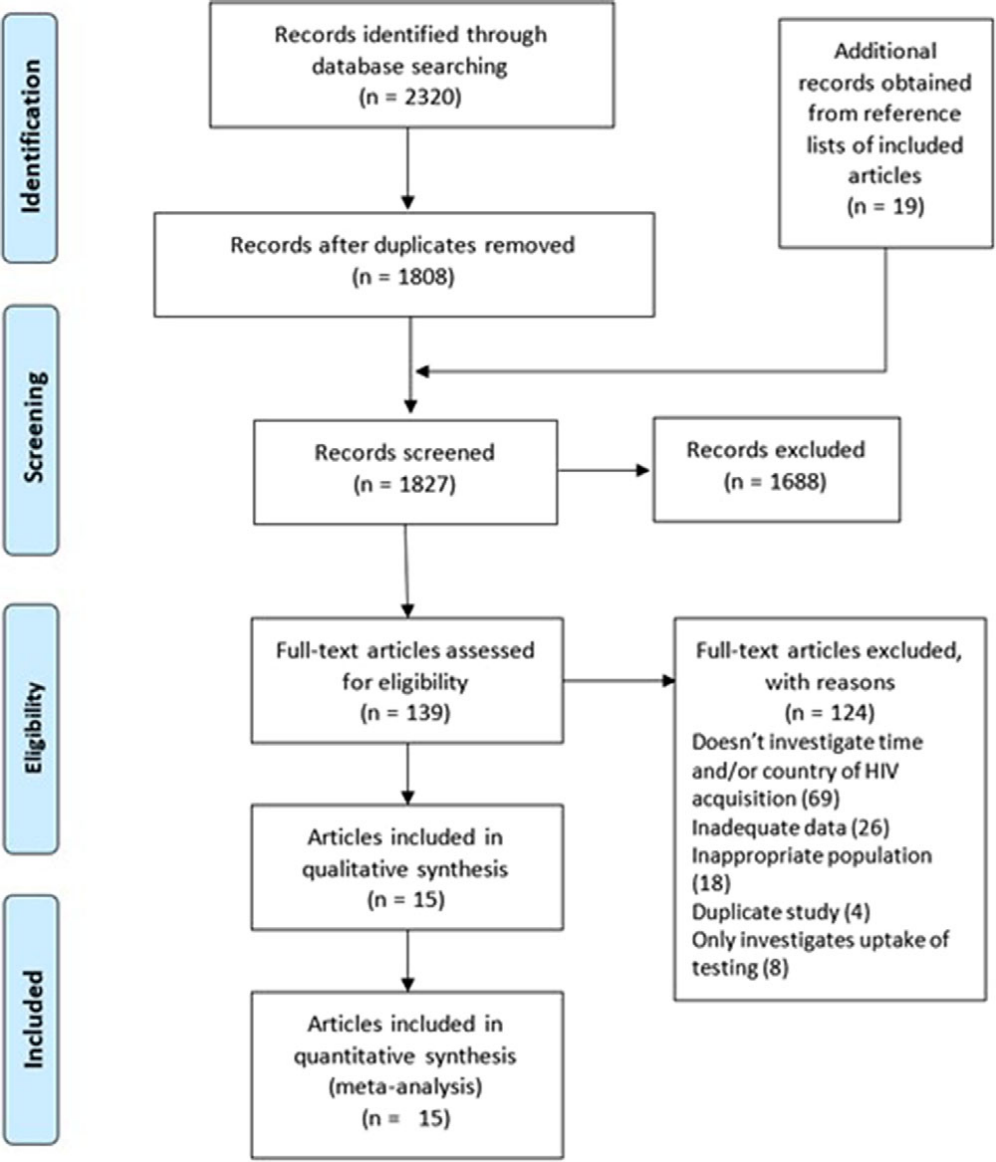
PRISMA flowchart of process for final inclusion of articles.

Titles and abstracts were screened independently by authors SM and ZM, and discrepancies were resolved by author RE. PRISMA guidelines were followed throughout this review (Figure 1). Following this, a full paper screen was performed. A quality assessment using the Newcastle–Ottawa Scale was undertaken for each paper that was included (Supplementary materials Table 3: *Quality Assessment).* To eliminate selection bias, all papers were included in analysis regardless of quality. Where we retrieved more than one article using the same data (such as the aMASE study or the PARCOURS study), we included the most recent article from the respective study groups.

### Data extraction and analysis

Key information was extracted by author SM, including reporting country, method of classifying PMHA, number of migrants in the study and number of migrants who acquired HIV post-migration. Studies were classified by reporting country, gender, sexuality, region of migrant origin, and method of PMHA classification, where available. Where exact numbers were not provided, we calculated figures for numbers of migrants with PMHA using the provided total number of migrants and proportions of PMHA.

Binomial proportions and standard errors were initially calculated for overall PMHA proportion for each study. We used the metaprop function in R to undertake a generalized linear mixed-effects model (GLMM) analysis by first fitting a logistic regression model to our extracted data and using maximum-likelihood to estimate τ^2^ [7]. This is equivalent to applying a random-effects model for meta-analysis as the mixed-effects model contains an intercept, with the random effect connected to that intercept using a binomial logit-link. This model is the recommended method for meta-analysis of proportions [8].

GLMM meta-analysis was performed across all studies and subgroup analysis was undertaken by (a) reporting country, (b) region of origin, and (c) gender and sexuality, to estimate a weighted average for overall PMHA and PMHA by subgroup. Clopper–Pearson 95% confidence intervals (CIs) were calculated for each study estimate and pooled average estimates of PMHA. CIs for these results were capped at 0% and 100% for presentation of pooled estimates as percentages. I^2^ statistic was calculated to quantify the extent of between-study heterogeneity in testing PMHA estimates. Analyses were performed in R, using the ‘metagen’ and ‘metabin’ functions in ‘meta’ [9, 10].

## Results

From our initial search, which included additional records from reference lists, 2,320 publications were retrieved. After removal of duplicates, this was reduced to 1808. After preliminary abstract screening, 139 papers underwent full paper screening. Fifteen were included for final analysis (Table 1) [6, 11–24]. Fourteen of the papers were retrospective cohort or cross-sectional studies, whilst one was a prospective cohort study [21]. Six studies identified PMHA using clinical reporting, whilst nine studies used modelling of CD4 count or another biomarker. We found the included studies appropriate for quantitative pooling using meta-analysis.

**Table 1.** Characteristics of study population for included studies

Source^a^	Country of study	Study type^b^	Migrant population			
			Ethnicity	Region of origin	Sexuality/gender	Other
Aggarwal et al. 2006	UK	Cross-sectional	Black African or Black Caribbean	Africa, Caribbean	Male only/Homosexual/bisexual, Heterosexual	—
Brannstrom et al. 2016	Sweden	Retrospective cohort	—	Continents: SSA, Asia/Pacific, L. America, Europe, Asia etc	Male, Female/Heterosexual, MSM	PWID, blood products
Brannstrom et al. 2017	Sweden	Retrospective cohort	—	African countries, Other	Male, Female/Heterosexual, MSM	PWID, Other
Desgrees-du-Lou et al. 2015	France	Retrospective cohort	SSA	Western, Central or Eastern/Southern Africa	Male, Female	Education level, social hardship
Dougan et al. 2004	England, Wales & Northern Ireland	Retrospective cross-sectional	Black Caribbean, Other	Caribbean, Other	Heterosexual male, Heterosexual female, MSM	—
Dougan et al. 2005	England & Wales	Retrospective cross-sectional	Black African, Black Caribbean	Caribbean, SSA	Male/MSM	—
Gras et al. 1999	Netherlands	Prospective cohort	Surinamese, Antillean, SSA	Ghana, Nigeria, Surinam, Dutch Antilles	Male, Female	—
Manfredi et al. 2001	Italy	Case–control matching	—	22 Countries in Africa (13), Europe (5), S. America (2), Asia (2)	Male, Female	PWID, congenital
Pantazis et al. 2019	Belgium, France, Germany, Greece, Italy, the Netherlands, Portugal, Spain, Switzerland, UK	Retrospective cross-sectional	—	Europe, Africa, Asia, S America	Male, Female	
Pantazis et al. 2021	EU & EEA countries	Retrospective cohort	—	SSA, W Europe, S Asia, Latin America, C Europe, Caribbean, Northern Africa & Middle East, E Europe, E Asia-Pacific, Australia & NZ	Male, Female, MSM, heterosexual	PWID
Paraskevis et al. 2017	Greece	Cross-sectional	—	—	—	PWID only
Rice et al. 2012	England, Wales & Northern Ireland	Cross-sectional	Black African, Black Caribbean, White, Other	Africa, Caribbean, Asia, Europe	Male, Female	—
Staehelin et al. 2004	Switzerland	Retrospective cohort	—	SSA, SE Asia, S Europe	Male, Female/Heterosexual, MSM	IVDU, Other
Stirrup et al. 2022	UK	Retrospective cohort	—	Europe, Africa	Heterosexual male, heterosexual female, MSM	
Yin et al. 2021	UK, Belgium, Sweden, Italy	Retrospective cohort	—	Europe, Africa, Caribbean + Latin America, North America, Asia-Pacific	Male, Female, heterosexual male, heterosexual female	

Abbreviation: SSA, Sub-Saharan Africa; UK, United Kingdom.

a See References for full citations.

b All are observational (cohort or cross-sectional) studies.

**Figure 2.**
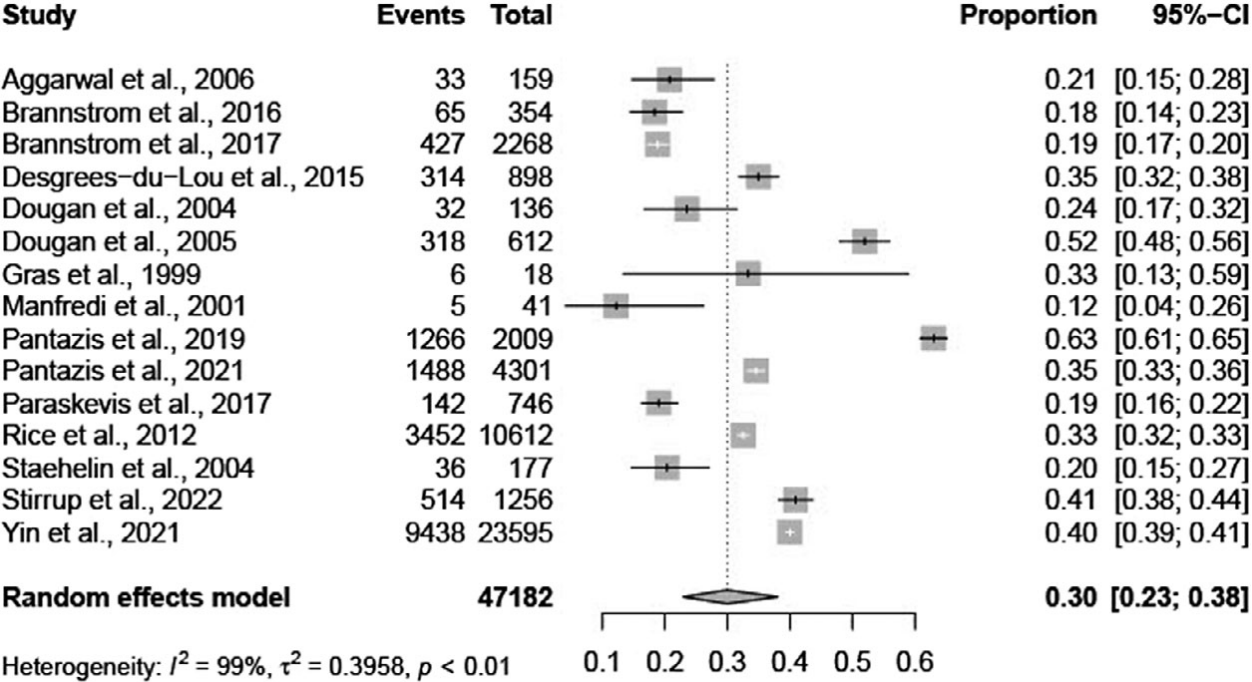
Forest plot of individual article and pooled average estimates for PMHA.

The total pooled average proportion of PMHA was 0.30 (95% CI: 0.23–0.38; Figure 2). The individual results are summarized below in Table 2. The total pooled average proportion of PMHA was based on 47,182 migrants in 11 European countries. The I^2^ test statistic was 99%, which indicates substantial heterogeneity between studies and the τ^2^ was 0.40. The observed proportions of PMHA in individual studies ranged from 0.12 (95% CI: 0.04–0.26) to 0.63 (95% CI: 0.61–0.65) [22,23].

**Table 2.** Total participants and method of PMHA ascertainment in studies included

Source	Study population (no. of migrants)	No of migrants who acquired HIV post-migration	PMHA estimate using generalized linear mixed-effects model (95% CI)^a^	Method of determining PMHA	Study quality
Aggarwal et al. 2006	159	33	0.21 (0.15–0.28)	Clinical records	5
Brannstrom et al. 2016	354	65	0.18 (0.14–0.23)	Clinical records	9
Brannstrom et al. 2017	2,268	427	0.19 (0.17–0.20)	Model	9
Desgrees-du-Lou et al. 2015	898	(not reported)	0.35 (0.32–0.38)	Model	7
Dougan et al. 2004	136	32	0.24 (0.17–0.32)	Clinical records	5
Dougan et al. 2005	612	318	0.52 (0.48–0.56)	Clinical records	6
Gras et al. 1999	18	6	0.33 (0.13–0.59)	Clinical records	5
Manfredi et al. 2001	41	5	0.12 (0.04–0.26)	Clinical records	5
Pantazis et al. 2019	10,612	3,452	0.63 (0.61–0.65)	Model	9
Pantazis et al. 2021	177.2	36	0.35 (0.33–0.36)	Model	9
Paraskevis et al. 2017	746	142	0.19 (0.16–0.22)	Model	6
Rice et al. 2012	23,595	9,438	0.33 (0.32–0.33)	Model	9
Staehelin et al. 2004	(not reported)	(not reported)	0.20 (0.15–0.27)	Model	8
Stirrup et al. 2022	4,301	1,488	0.41 (0.38–0.44)	Model	8
Yin et al. 2021	2009	(not reported)	0.40 (0.39–0.41)	Model	9

a If PMHA was given as a range, or in studies which produced multiple estimates, the most conservative (i.e., the lowest) estimate was used.

### Host country

Five studies were based in the UK, two in Sweden, one in France, one in Greece, one in Italy, one in the Netherlands, and one in Switzerland. The remaining three studies reported on multiple countries [18, 19, 24]. Of these three studies, Panties et al. and Yin et al. both provided disaggregate numbers for PMHA within several ‘host’ countries, and these data are used where possible [29].

**Figure 3.**
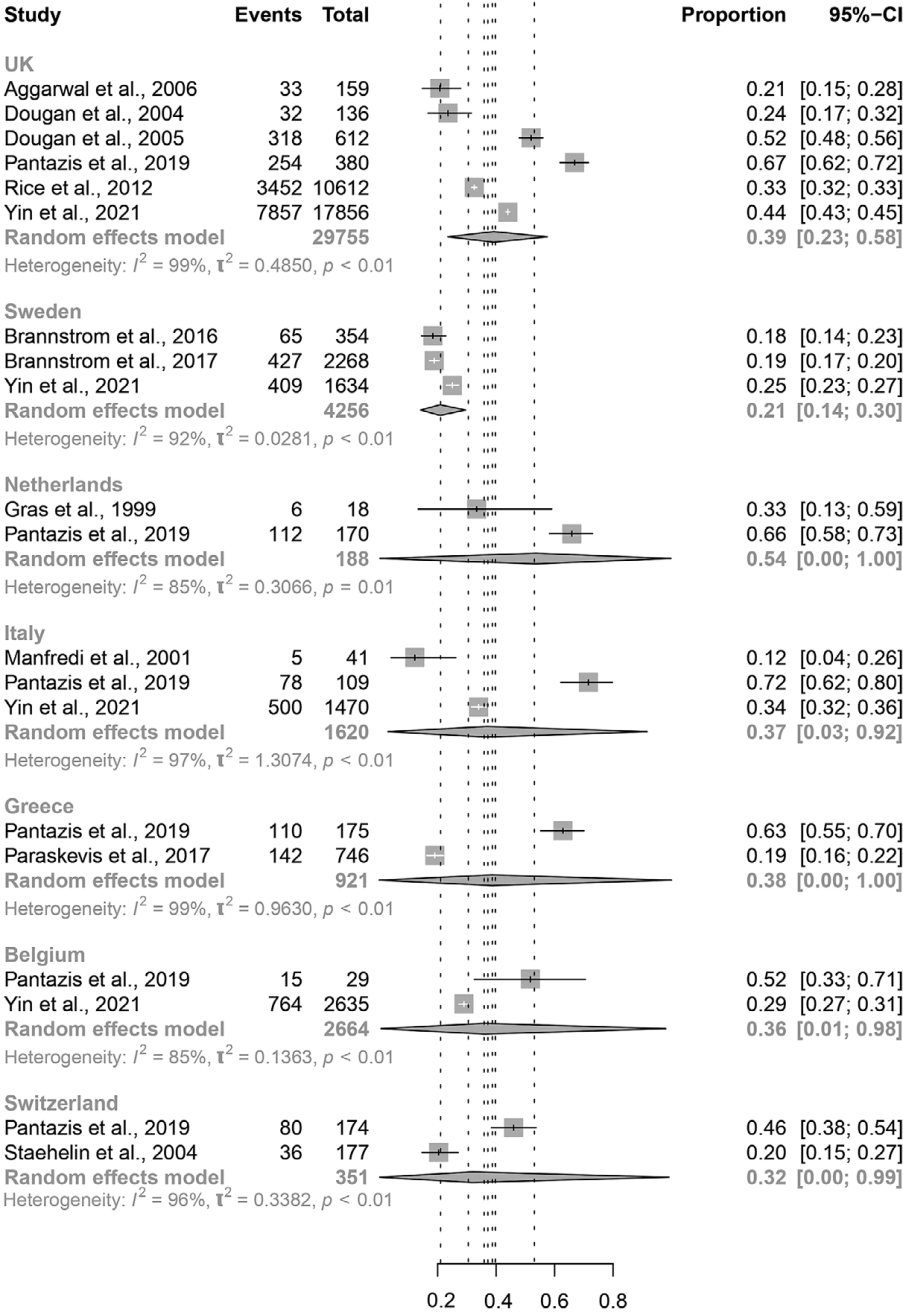
Forest plots for individual and pooled average PMHA estimates by host country.

Our findings show no significant difference in proportion of PMHA between host countries (Figure 3). The greatest proportion of PMHA is seen in the Netherlands (0.54, 95% CI: 0.00–1.00), and the lowest is seen in Sweden (0.21, 95% CI: 0.14–0.30). The three papers which presented data estimating the level of PMHA in Sweden all found similar results, with one article using clinicians’ records and the others using modelling [12, 24].

### Region of origin

**Figure 4.**
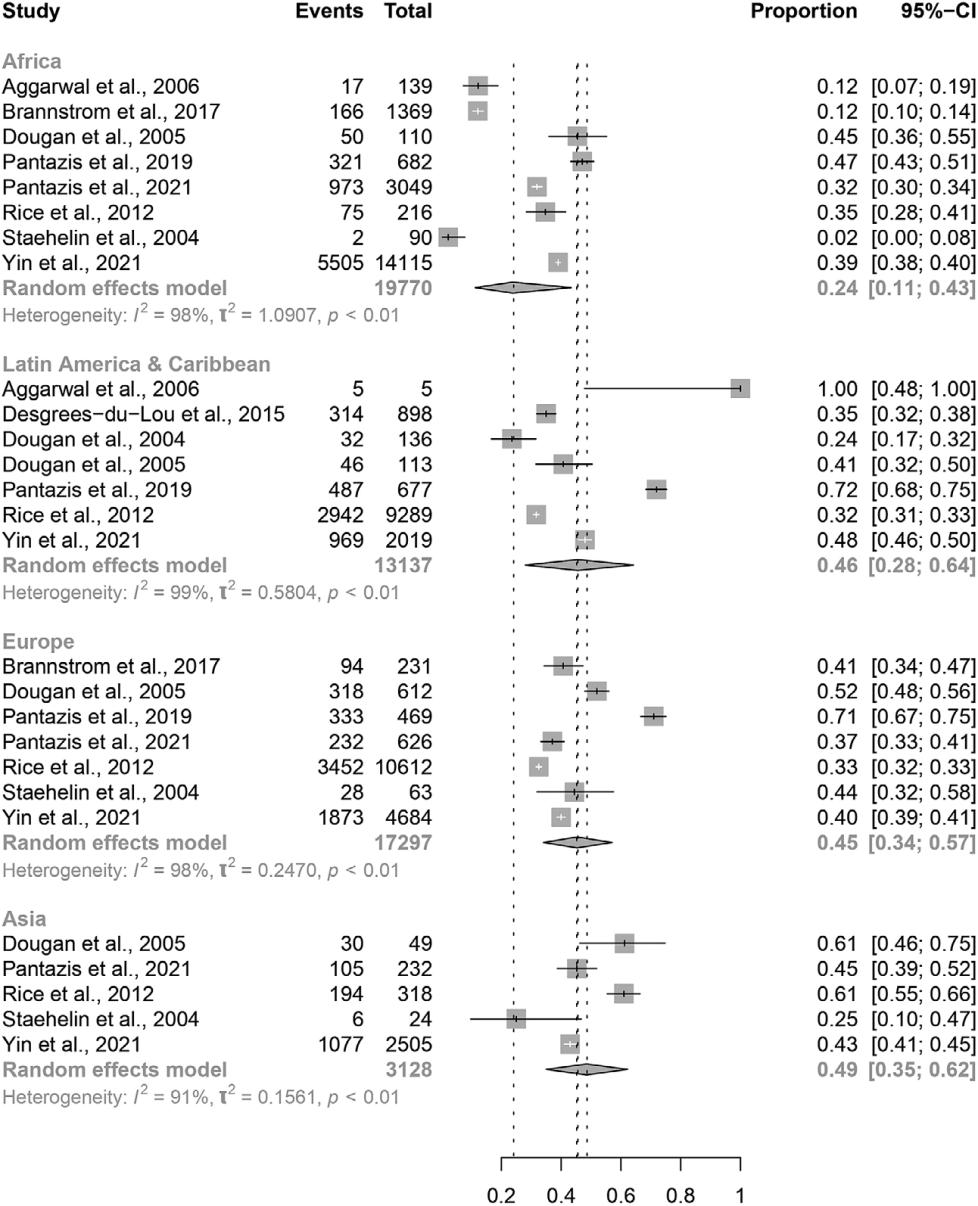
Forest plots for individual and pooled average PMHA estimates by migrant region of origin.

Eight papers included migrants from Africa [11, 12, 14, 18, 19, 21, 22, 24]; seven from Latin America and Caribbean [6, 11, 14, 15, 18, 21, 24]; and seven studies from Europe [12, 14, 18, 19, 21, 22, 24]. Five papers were included from Asia [14, 19, 21, 22, 24]. Data from South/Latin America were combined with data from the Caribbean as these studies included migrants from regions with a similar background prevalence of HIV [25].We found no significant difference in the estimated proportion of PMHA between the four regions of origin for migrants in Europe (Figure 4). Estimated proportion of PMHA was lowest in migrants from Africa (0.24, 95% CI: 0.11–0.43) and highest in migrants from Asia (0.49, 95% CI: 0.35–0.62).

### Gender and sexuality

**Figure 5.**
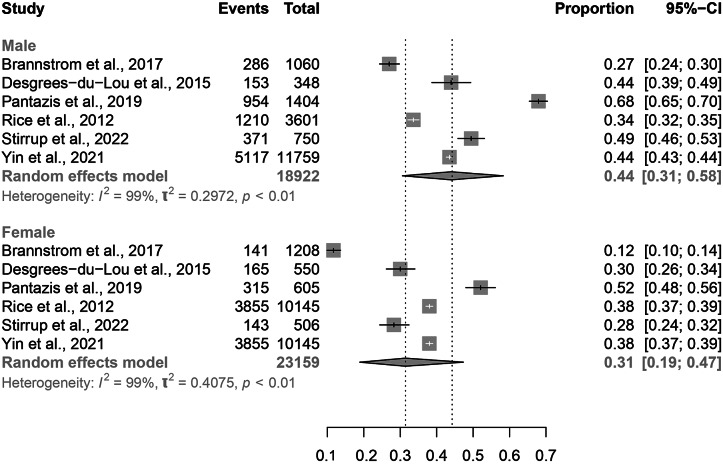
Forest plots for individual and pooled average PMHA estimates by migrant sex.

**Figure 6.**
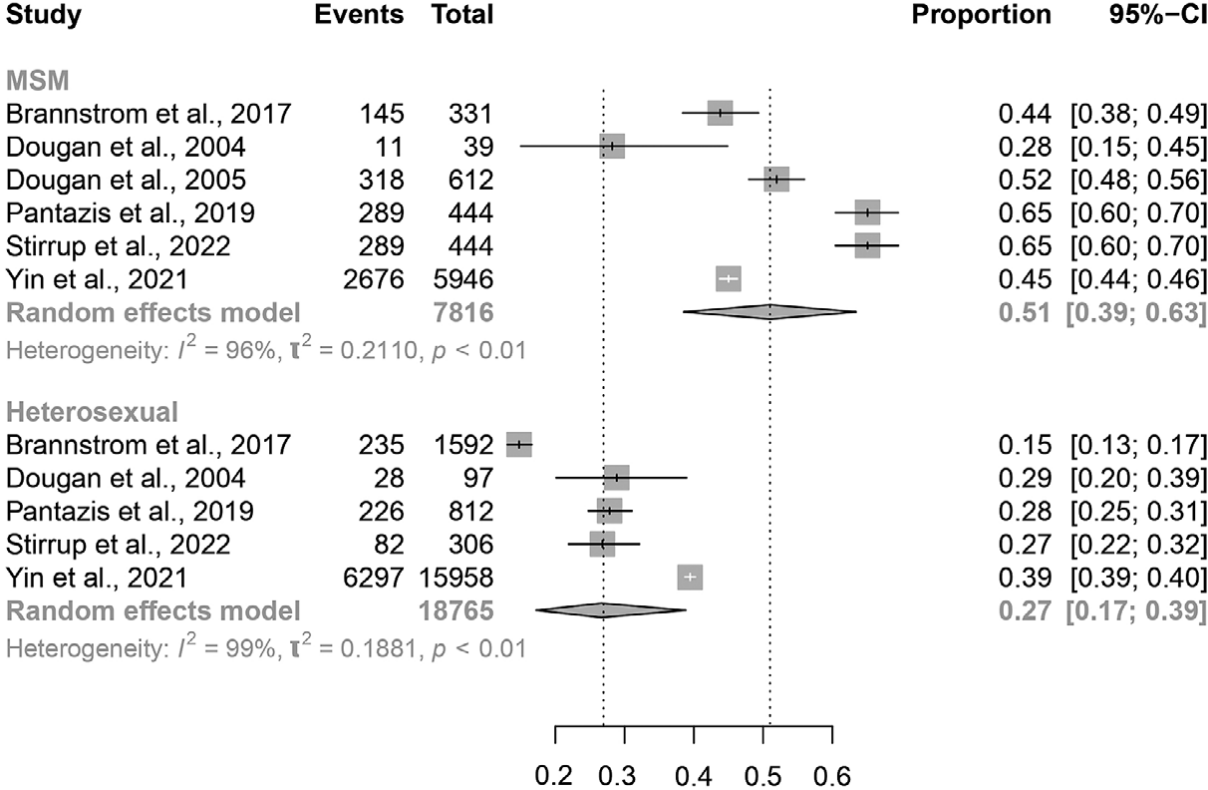
Forest plots for individual and pooled average PMHA estimates by migrant sexuality.

Eight studies reported data on gender and sexuality [6, 12, 14, 15, 18, 21, 23, 24]. Of these, six included migrant MSM and five included heterosexual migrants. There was no significant difference in proportion of PMHA between males (0.44, 95% CI: 0.31–0.58) and females (0.31, 95% CI: 0.19–0.47), although the overlap in confidence intervals between these two groups is small (Figure 5). There was a borderline significant difference in estimated proportion of PMHA between MSM migrants (0.51, 95% CI: 0.39–0.63) and heterosexual migrants (0.27, 95% CI: 017–0.39; Figure 6).

### Method of PMHA classification

**Figure 7.**
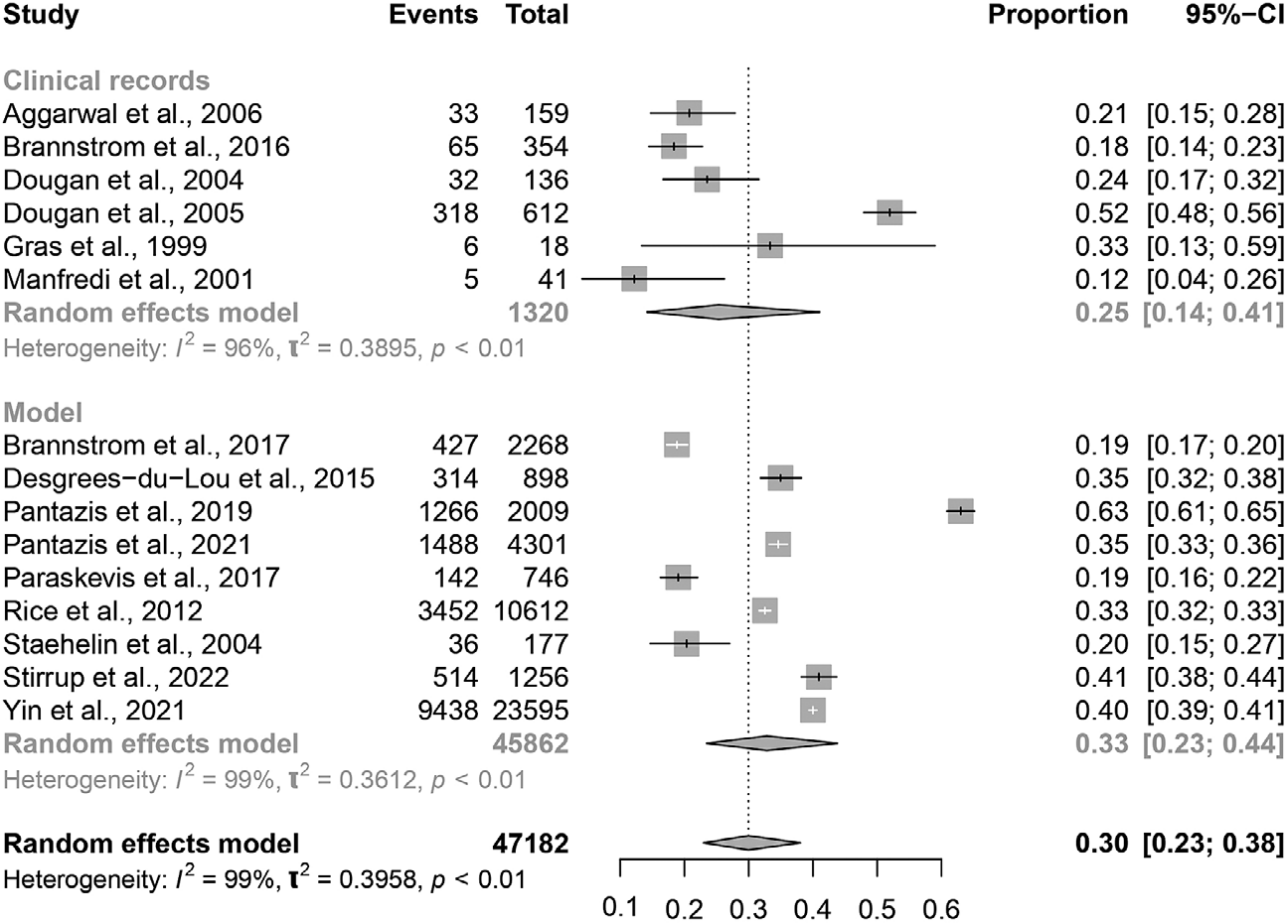
Forest plots for individual and pooled average PMHA estimates by method of PMHA classification.

Studies which classified PMHA by clinical records describe using either self-reporting or evidence of a previous negative HIV test in the host country; however, Gras et al. also used proxies such as being sexually active only after migration [21]. The proportion of PMHA in these studies ranged from 0.12 (95% CI: 0.14–0.41; Manfredi et al. 2001) to 0.52 (95% CI: 48–0.58) [19,22]. The forest plots for individual and pooled average PMHA estimates by method of PMHA classification are seen in Figure 7.

Nine articles used modelling to estimate PMHA: eight of these used CD4 count or viral load, either alone or in conjunction with clinical notes, whilst Paraskevis et al. investigated local transmission networks and then estimated country of infection based on behavioural/clinical data and phylogenetic analysis of strains [25]. The lowest proportion of PMHA among these studies was seen in Brannstrom et al. and Paraskevis et al. (0.19, 95% CI: 0.17–0.20; 0.19, 95% CI: 0.16–0.22), and the highest proportion was seen in Pantazis et al. 2019 (0.63, 95% CI: 0.61–0.65) [18]. There was no significant difference in proportion of PMHA between studies using clinical records (0.25, 95% CI: 0.14–0.41) and studies using models (0.33, 95% CI: 0.23–0.44).

## Discussion

In this systematic review and meta-analysis, we aimed to assess the quantity of post-migration HIV acquisition (PMHA) in migrants to European countries. Although there have been some significant cohort studies assessing this, to our knowledge this is the first systematic review and meta-analysis of all the available data. We identified a total of fifteen studies and found the overall average proportion of PMHA across Europe was 30% (95% CI: 23–38%). This finding varied based on host country, region of origin, sex, sexuality, and method of classifying PMHA. The highest overall proportion was seen in Pantazis et al. (63%), and the lowest was seen in Manfredi et al. (12%) [22,23].

The variation seen in host country PMHA, from 0.21 (95% CI: 0.14–0.30; Sweden) to 0.54 (95% CI: 0.00–1.00; Netherlands), could reflect country-level differences in services for HIV prevention, diagnosis, and treatment. For example, PMHA was lowest in Sweden, thus indicating that HIV care is perhaps more accessible and effective in Sweden compared with other countries. Our results may reflect a lower rate of high-risk behaviours among migrant populations Sweden. Alternatively, countries with a low PMHA, such as Sweden and Switzerland, may represent migrant populations with a higher level of pre-migration HIV acquisition due to a higher background prevalence of HIV, reducing post-migration HIV acquisition levels [26].

The very large confidence intervals for overall PMHA in Netherlands, Italy, Greece, Belgium, and Switzerland reflect the heterogeneity of data within host countries and across time periods. For example, the proportion of PMHA in the Netherlands is 0.33 (95% CI: 0.13–0.59) according to Gras et al. in 1999, whereas in 2019 Pantazis et al. found that the proportion of PMHA in the Netherlands is 0.66 (95% CI: 0.58–0.73) [21,24]. This should be interpreted with caution in view of Gras et al.’s small sample size; the difference may reflect a true higher rate of PMHA in the Netherlands after 20 years, but it is likely also the result of better surveillance in 2019 compared with 1999.

As with analysis of other subgroup characteristics, our results for host countries are limited by factors related to differing migrant demographics, which may contribute to the variation in estimates seen for each country. For example, some of the UK papers only reported on Black African migrants, reducing the accuracy of our result for average PMHA in all UK migrants. Several countries lacked sufficient data for subgroup analysis, whereas the UK may be over-represented in this review, with five UK papers included. This suggests good clinical practice in the UK of recording estimated time/country of HIV infection. However, it could be due to a language bias in our search, as search terms were in English.

Migrants from African countries had a non-significantly lower proportion of PMHA than migrants from Europe, Latin America, Caribbean, and Asia. African migrants are more likely to come from high-prevalence countries, so a larger proportion may already have HIV at the time of migration; this would reduce the number of African migrants who are HIV-negative on entry and therefore at risk of HIV acquisition post-migration. However, a lower level of PMHA as a proportion of all HIV cases in migrants does not suggest a low overall number of African migrants acquiring HIV post-migration. Black African patients bear a large proportion of the burden of HIV in Europe, with migrants originating in sub-Saharan Africa representing 18% of HIV diagnoses in Europe in 2019 [27]; if 24% of these cases could have been acquired in the host country, this is nonetheless a significant number in the context of HIV diagnoses in Europe. This review included more migrants from Africa (n = 30,458) than from Europe (n = 7,088), so our findings may have been influenced by disproportionate sampling.

The higher estimate for average PMHA in migrants from Asia, Latin America, Caribbean, and other European countries indicates that preventing HIV in these migrants should also be a public health priority in the host country. We did not have individual-level data to comment on which European countries migrants originated from, nor whether acquisition rates were comparable with that in the host countries’ native populations, both of which might help to explain the result.

### Gender and sexuality

Although not statistically significant, the higher proportion of PMHA in MSM migrants when compared with heterosexual migrants likely reflects greater risk of HIV acquisition post-migration among MSM. Further research with a larger volume of individual-level data could directly compare PMHA in MSM with that in heterosexuals. Furthermore, more specific target groups for HIV prevention programmes could be identified by exploring PMHA among MSM migrants within the subgroup of region of origin. Of note, there were no statistically significant differences in the average PMHA among the subgroups of heterosexual migrants (total women, total men, heterosexual men), which is in keeping with the epidemiology of HIV generally and of that observed among Australian migrants post-migration [28].

### Classifying PMHA

Average PMHA was higher when using CD4 or other biomarker modelling articles (0.33, 95% CI: 0.23–0.38) than average PMHA using clinical records (0.25, 95% CI: 0.14–0.41). Rice et al. and Brannstrom et al. estimated PMHA using ‘Life events’ and CD4 decline modelling separately, before comparing the two sets of outcomes; both studies confirmed that clinicians estimated a lower proportion of PMHA than mathematical models [26,27]. This may be due to risk of bias from self-reporting or in clinical history-taking. Using clinicians’ notes, we assumed that there was PMHA only if the country of acquisition was recorded as the reporting country. However, if the clinician suspected HIV was acquired during a trip abroad taken after migration, the infection would be recorded as acquired ‘outside of the country of residence’; using this data, we would falsely classify this case as ‘not PMHA’. Thus, clinical data likely underestimated the proportion of PMHA [34]. Migrants travelling to their country of origin could be at increased risk of acquiring HIV post-migration and this therefore presents a public health priority for host countries [35,36].

CD4 modelling has been credited as a reliable method of producing an estimate of time of infection and as such, estimates from these papers are considered as the most reliable in this review. However, rate of CD4 cell decline is influenced by age, ethnicity, comorbidities, and strain of HIV; not all studies adjusted for these variables, which was considered in assessment of study quality [37,38].

The level of HIV acquisition depends on a multitude of factors that were not analysed in this review, including cultural practices, education level, and socio-economic status [39–43]. Some of these factors are likely to help explain the variation in PMHA found in this review. Demographic information about migrants with PMHA could inform future, targeted prevention interventions to reduce HIV transmission; this review calls for further research to provide individual-level demographic data.

In order to accurately define PMHA, multiple studies retrieved in our search, including high-quality articles which used data from the aMASE study or the PARCOURS study, had to be excluded to avoid duplicate data with Pantazis et al. 2019 and Desgrees du-Lou et al. 2015, respectively [10,11,23,44,45].

In order to strengthen consistency, studies were also excluded from our analysis if the authors’ definition of migrant included second-generation migrants [46]. Among these was a 2013 ECDC report on HIV transmission in migrants within Europe: PMHA estimates in this report varied from 0.02–0.62 and authors found that PMHA was lower in migrants from Africa than those from Asia or the Caribbean, corresponding with our findings [47].

### Limitations

The main limitation for this study was heterogeneity in the articles included in analysis. Although we were able to undertake meta-analysis, variation in study design and migrant populations demographics, as well as method for PMHA classification, resulted in high I^2^ statistics, indicating considerable heterogeneity in pooled results. Nonetheless, we have provided the most reliable estimate utilizing the most accurate data currently available. PMHA could be further investigated with more individual-level data and further information on factors that may have a confounding impact (e.g., migrant demographics, age, duration of residence in the reporting country, etc.)

Within subgroups, estimates varied, due to the relative smaller sample sizes and between study heterogeneity. Migrants are not a homogeneous group and thus fall into a variety of differing demographics, cultural practices, ideologies, and ultimately behaviours that cannot be simply classified or retrieved or adjusted for in our analysis. The heterogeneity observed is also likely due in part to changes in the level of PMHA over time. Data from the aMASE study are more current and show a higher average PMHA than other articles, so their results could indicate a recent increase in the proportion of PMHA in several reporting countries [23].

Further research is needed to find an accurate proportion of PMHA for different European countries or regions based on their unique profiles, that is, country demographics, health service structure, and patterns of migration. With better surveillance of PMHA in individual countries and across Europe, estimates could be compared with HIV risk for the native populations. This would not only allow for a far better understanding of the complex factors contributing to the risk of PMHA on an individual migrant level, but also allow for better public health planning.

## Conclusions

This review provides the most reliable estimate of PMHA based on the current literature. Our findings suggest that migrants continue to be at high risk of HIV acquisition in Europe. This indicates the need for targeted screening and HIV prevention interventions, ensuring resources are appropriately directed to combat the spread of HIV.

## Supporting information

Mann et al. supplementary material Mann et al. supplementary material

## Data Availability

The data that support the findings of this study are openly available in the references listed.

## Supplementary material

For supplementary material accompanying this paper visit http://doi.org/10.1017/S0950268824000372. click here to view supplementary material
