# Supplementary material for: Post-migration HIV acquisition: A systematic review and meta-analysis
Source: Epidemiol Infect. 2024 Mar 1;152:e49. doi: 10.1017/S0950268824000372 (PMC11022255; doi:10.1017/S0950268824000372)
Supplement: Mann et al. supplementary material [file S0950268824000372sup001.pdf]

Supplementary materials

Table 1 - Search terms

	**Search**
1	exp migrant/
2	refugee*.mp. [mp=title, abstract, heading word, drug trade name, original title, device manufacturer, drug manufacturer, device trade name, keyword, floating subheading word, candidate term word]
3	migrant*.mp. [mp=title, abstract, heading word, drug trade name, original title, device manufacturer, drug manufacturer, device trade name, keyword, floating subheading word, candidate term word]
4	(displaced adj (person* or people*)).mp. [mp=title, abstract, heading word, drug trade name, original title, device manufacturer, drug manufacturer, device trade name, keyword, floating subheading word, candidate term word]
5	immigrant*.mp. [mp=title, abstract, heading word, drug trade name, original title, device manufacturer, drug manufacturer, device trade name, keyword, floating subheading word, candidate term word]
6	asylum.mp. [mp=title, abstract, heading word, drug trade name, original title, device manufacturer, drug manufacturer, device trade name, keyword, floating subheading word, candidate term word]
7	1 or 2 or 3 or 4 or 5 or 6
8	exp Human immunodeficiency virus/
9	exp Human immunodeficiency virus infection/
10	exp Human immunodeficiency virus infection/
11	8 or 9 or 10
12	exp diagnosis/
13	11 and 12
14	((hiv or hiv1 or hiv2 or aids or human immunodeficiency virus or human immunedeficiency virus or human immune* deficiency virus or acquired immunodeficiency syndrome or acquired immunedeficiency syndrome or acquired immune* deficiency syndrome) adj3 (diagnos* or test or tests or testing)).mp. [mp=title, abstract, heading word, drug trade name, original title, device manufacturer, drug manufacturer, device trade name, keyword, floating subheading word, candidate term word]
15	13 or 14
16	(Europ* or EU or Albania or Andorra or Armenia or Austria or Azerbaijan or Belarus or Belgium or Bosnia or Herzegovina or Bulgaria or Croatia or Cyprus or Czechia or Denmark or Estonia or Finland or France or Georgia or Germany or Greece or Hungary or Iceland or Ireland or Italy or Kazakhstan or Kosovo or Latvia or Liechtenstein or Lithuania or Luxembourg or Malta or Moldova or Monaco or Montenegro or Netherlands or Macedonia or Norway or Poland or Portugal or Romania or Russia or 'San Marino' or Serbia or Slovakia or Slovenia or Spain or Sweden or Switzerland or Turkey or Ukraine or 'United Kingdom' or UK or Britain or England or 'Vatican City').mp. [mp=title, abstract, heading word, drug trade name, original title, device manufacturer, drug manufacturer, device trade name, keyword, floating subheading word, candidate term word]
17	exp Europe/
18	16 or 17
19	7 and 15 and 18
**20**	**limit 19 to yr="1996 -Current"**

Table 2 - Screening criteria

Stage	Inclusion criteria	Exclusion criteria
**Title and abstract screen:**	• Population: First generation, long term migrants (UN definition)<br>• Intervention:<br>○ HIV testing (CD4 count, viral load) on two separate occasions post-migration.<br>○ Trajectory modelling and analysis based on time of migration and CD4 count at diagnosis<br>• Comparator: Migrants who acquired HIV before migration or do not have a previous HIV -ve test<br>• Outcomes:<br>○ Proportion of migrants who have a positive HIV test with a previously documented or self-reported negative HIV test.<br>○ Proportion of migrants who are found to have acquired HIV post-migration, based on CD4 count modelling<br>○ Quantitative data on HIV prevalence in migrants<br>• Study features<br>○ Study published from 1996 onwards<br>○ Observational studies, surveillance studies, mathematical modelling	• The study population is not in Europe<br>• The paper does not focus on migrants<br>• The paper only investigates HIV testing<br>• The paper does not investigate or quantify HIV prevalence<br>• The paper has an irrelevant intervention or outcome

Table 3 – Quality assessments (Newcastle-Ottawa Scale)

**Criterion**	**Acceptable (1 star awarded)**	**Unacceptable (no stars awarded)**
Representativeness of exposed cohort	Population-based data on all migrants selected from multiple regions/areas	Hospital/Clinic-based data from tertiary care or focused on a specific migrant group (e.g. only MSM) or selected from a single region/area
Ascertainment of exposure	Confirmed HIV positive + born outside of host country.	Study population is based on ethnicity (but provides data based on region of birth, otherwise exclude).
Demonstration that outcome of interest was not present at start of study	Negative tests pre/post-migration or model estimate	Unclear clinical evidence or unreliable model used
Comparability of cohorts	1 *s: Migrants selected from same area/region in host country + same time period<br>2 *s: As above PLUS model uses migrants who are matched/stratified by 2 or more of: age, gender, socioeconomic status	Compared with model recruited from separate dataset with no matching for time + location
Assessment of outcome	Used a reliable method to assess time/place of acquisition (clinical records, modelling).<br>Excluded 'unclear country of acquisition' or used lower estimate of PMHA	Any lack of clarity in method of estimating PMHA or final estimate did not exclude the 'unclear' cases.<br>More than 30% cases could not be classified as pre or post migration
Length of follow-up	Migrants who have been living in the country for at least 1 year	Migration less than 1 year ago
Adequacy of follow-up	Excluded cases which do not meet the above criteria	-

Study	Assessment of quality of a cohort study – Newcastle Ottawa Scale Domain										Method of PMHA classification
	Selection				Comparability	Outcome					
	1. Representativeness of the exposed cohort	2. Ascertainment of exposure	3. Demonstration that outcome of interest was not present at start of study	Total (max. 3)	Comparability of cohorts on the basis of the design or analysis (max. 2)	1. Assessment of outcome (max. 2)	2. Was follow-up long enough for outcomes to occur?	3. Adequacy of follow-up of cohorts	Total (max. 4)	Total NOS Score (max. 9)	
Aggarwal et al. 2006	0	0	*	1/3	*	*	*	*	3/4	**5/9**	Clinical records / Self reporting
Brannstrom *et al* 2016	*	*	*	3/3	*	**	*	*	4/4	**9/9**	Clinical records / Self-reporting
Brannstrom *et al* 2017	*	*	*	3/3	**	**	*	*	4/4	**9/9**	CD4 decline model
Desgrees-du-Lou *et al* 2015	*	*	*	3/3	*	**	*	*	4/4	**7/9**	CD4 decline model
Dougan *et al* 2004	0	0	*	1/3	*	*	*	*	3/4	**5/9**	Clinical records / Self-reporting
Dougan *et al* 2005	0	*	*	2/3	*	*	*	*	3/4	**6/9**	Clinical records / Self-reporting
Gras *et al* 1999	0	0	*	1/3	*	*	*	*	3/4	**5/9**	Clinical records / Self-reporting
Manfredi *et al* 2001	0	*	0	1/3	**	*	*	*	3/4	**5/9**	Clinical records / Self-reporting
Paraskevis *et al.* 2017	0	0	0	1/3	**	*	*	*	3/4	**6/9**	Phylogenetic analysis
Rice *et al* 2012	*	*	*	3/3	**	**	*	*	4/4	**9/9**	CD4 decline model
Staehelin *et al* 2004	*	*	*	3/3	*	**	*	*	4/4	**8/9**	CD4 decline model
Yin *et al* 2021	*	*	*	3/3	**	**	*	*	4/4	**9/9**	CD4 decline model

**Stirrup *et al* 2022**	0	*	*	2/3	**	**	*	*	4/4	**8/9**	Phylogenetic analysis + Bayesian model
**Pantazis et al 2019**	*	*	*	3/3	**	**	*	*	4/4	**9/9**	Biomarker decline + Bayesian model
**Pantazis et al 2021**	*	*	*	3/3	**	**	*	*	4/4	**9/9**	CD4 (+viral load) decline
*** Modified Newcastle-Ottawa Scale score: Selection (max.***); Comparability (max.**); Outcome (max.****)**											
